# A CBCT Assessment of Apical Transportation in Root Canals Prepared with Hand K-Flexofile and K3 Rotary Instruments

**Published:** 2014-12-24

**Authors:** Zahra Sadat Madani, Daryoush Goudarzipor, Azam Haddadi, Akam Saeidi, Ali Bijani

**Affiliations:** a *Department of Endodontics, Dental School, Babol University of Medical Sciences, Babol, Iran; *; b * Department of oral and Maxillofacial Radiology, Dental School, Tehran University of Medical Sciences, Tehran, Iran *; c * Department of Endodontics, Dental School, Mazandaran University of Medical Sciences, Sari, Iran; *; d * Endodontist, Private Practice,Sari, Iran; *; e * Non-Communicable Pediatric Diseases Research Center, Babol University of Medical Sciences, Babol, Iran*

**Keywords:** Apical Transportation, Cone-Beam Computed Tomography, K3, K-Flexofile, Root Canal Therapy

## Abstract

**Introduction:** Apical transportation changes the physical shape and physiologic environment of the root canal terminus. The aim of the present experimental study was to determine the extent of apical transportation after instrumentation with hand K-Flexofile and K3 rotary instruments by means of cone-beam computed tomography (CBCT). **Methods and Materials:** Forty mesiobuccal root canals of maxillary first molars, with 19-22 mm length and 20-40^°^ canal curvature, were selected and assigned into two preparation groups. The first group was prepared with K-Flexofile with passive step-back technique and the second group was prepared with K3 rotary instruments. Pre and post instrumentation CBCT images were taken under similar conditions. The amount of root canal transportation was evaluated by Mann-Whitney U test and the chi-square test was used for the qualitative evaluation. **Results:** The amounts of apical canal transportation with the K3 and K-Flexofile instruments were 0.105±0.088 and 0.150±0.127 mm, respectively with no statistically significant differences. In the manual technique, 25% of the canals had no apical transportation; while 30% of the canals in the K3 group were transportation free. **Conclusion:** Both systems were able to preserve the initial curvature of the canals and both had sufficient accuracy. Preparation with K3 rotary instruments resulted in apical transportation similar to that of K-Flexofile.

## Introduction

One of the most important steps in root canal treatment is mechanical preparation, debriding the canal and creating a cone-shaped configuration for easy access, effective irrigation and three-dimensional obturation of the root canal space [1]. Regardless of the technique used for debridement, this procedure results in removal of root canal walls, to some extent. Removal of more dentin from one side compared to other side of the canal wall which are located at similar distances from the long axis of the root, results in a procedural error known as canal transportation [2]. Canal transportation results in displacement of the physiologic end of the canal to a new operator-made location on external surface of the root, leading to accumulation of residual debris and microorganisms [3]. Moreover, this procedural error compromises the uniformity of the root and reduces its fracture resistance and finally results in poor prognosis of treatment [4]. Continuing debridement of the transported path by larger files creates a tear drop appearance at the apical area of the canal and might result in lateral perforation of the root [5]. The shape created due to canal transportation does not provide a resistant form to condense gutta-percha which leads to poor compaction and over-extension of gutta-percha [3]. Deviation from the initial form of the root canal, especially in the apical area, prevents proper obturation and seal against bacterial penetration, which can potentially result in treatment failure.

Besides development of various techniques for the debridement of the root canal that could eliminate such problems, some modifications have been suggested in instruments design. Engine driven instruments can shorten the treatment sessions and reduce the practitioner and patient’s tiredness. However, these systems have some drawbacks including straightening of the root canal, absence of a tactile sensation, inadequate debridement and the risk of file fracture [6].

In 1982 Kerr manufacturing company introduced the K-Flexofile, a departure from the square and triangular configurations [7]. This special cross-section design presents significant changes in instrument flexibility and cutting characteristics. The cutting edges of the high flutes are formed by the two acute angles of the rhombus and present increased sharpness and cutting efficiency. The alternating low flutes formed by the obtuse angles of the rhombus are meant to act as an auger, providing more area for increasing the reservoir that, with proper irrigation, increases debris removal. The decreased contact between the instrument and canal walls provides a space for compacting dentinal filings within the canal [8].

The use of stainless steel hand files produces a high level of procedural errors. Introduction of nickel-titanium (NiTi) rotary instruments has provided easier and faster canal instrumentation and has minimized the procedural errors and operator fatigue [9] and enhanced the success rate of root canal treatment in comparison with stainless steel hand instruments [10]. K3 Endo NiTi rotary file system (Sybron Endo, Orange, CA, USA) was introduced in 2002. These files are designed with a wide radial land and slightly positive rake angle [11]. This file has a variable core diameter designed to increase its flexibility, and a safe-ended tip to decrease the incidence of ledging, perforation and zipping [11].

Recently, noninvasive techniques such as cone-beam computed tomography (CBCT) have been utilized to evaluate canal anatomy and shape before and after preparation at different levels [12]. At present, CT scanners have become a popular and routine technique in the evaluation of shaping ability of new endodontic systems due to their practicality and non-destructive nature [13-15]. CBCT images are highly accurate compared to conventional techniques and do not involve destruction of the specimen, have high reproducibility, and can provide several images from the canals [2, 16-18].

Considering the advantages of CBCT in evaluation of apical transportation [19], the aim of this experimental study was to compare apical transportation in root canals instrumented with hand K-Flexofile to K3 rotary instruments using CBCT technique.

**Table 1:** Mean (SD) of apical transportation values in two groups

**Group**	**N**	**Mean (SD)**	***P*** **-value**
**K-Flexofile**	20	0.150 (0.127)	0.20
**K3**	20	0.105 (0.088)	

## Materials and Methods

The research protocol was approved by the Ethics committee of Babol University of Medical Sciences, Babol, Iran (Grant No. 303150). In this study, 40 extracted human maxillary first molars with fully developed apices were obtained with the length of mesiobuccal (MB) canals being in the range of 19-22 mm and canal curvature being within 20 to 40 degrees, measured according to the Schneider’s method [20]. The teeth were kept in 0.1% thymol solution at 9^°^C for disinfection, and 24 h prior to the process, they were washed with running tap water to eliminate traces of thymol and were then stored in normal saline at 4^°^C. Standard straight-line access cavities were prepared and the MB canals were located. Then, for each tooth a #10 K-File (Dentsply Maillefer, Ballaigues, Switzerland) with RC-Prep (Premier Dental Products, Philadelphia, USA) was inserted into the MB canal until it became visible from the apical foramen. Subsequently, the working length was determined 1 mm short of this length to preserve the apical constriction. The teeth were mounted in dental stone blocks to facilitate instrumentation and to enable the reproducibility of pre- and post-operative CBCT images.

The teeth were randomly divided into two groups (*n*=20). The coronal segment of the canals was prepared using sizes 1 to 3 Gates-Glidden drills (Dentsply Maillefer, Ballaigues, Switzerland). In addition, 2 mL of 2.5% NaOCl was used during instrumentation of the canals in both groups.

***Group 1 (hand instrumentation)***

In this group, the MB canals were debrided with hand K-Flexofile (Dentsply Maillefer, Ballaigues, Switzerland) using the passive step-back technique. Debridement continued up to #30 file as the master apical file (MAF).

***Group 2 (rotary technique)***

In this group, the MB canals were debrided with K3 rotary file system (Sybron Endo, Orange, CA, USA) using an electric motor (Endo-Mate TC, NSK, Nakanishi Inc., Tokyo, Japan) set at a speed of 300 rpm and torque of a 2 Nm based on manufacturer’s instructions. Apical preparation was performed with files 25/0.10, 25/0.08, 40/0.04, 35/0.04 and 30/0.04. Each file series was discarded after use in one MB canal or when any defect or deformation was observed in the shape or structure of the file. Instrumentation was carried out by the same operator in both groups. The operator prepared 5 canals each day so that a constant and uniform force would be applied during canal preparation and operator fatigue would not exert any effect on the results.

**Table 2:** Frequency of apical transportations in the root canals prepared by K-Flexofile and K3 files

**Group**	**Apical transportation**		
	**Absent N (%)**	**Present N (%)**	**Total N (%)**
**K-Flexofile**	5 (25)	15 (75)	20 (100)
**K3**	6 (30)	14 (70)	20 (100)
**Total**	11 (27.5)	29 (72.5)	40 (100)

***Evaluation of transportation***

Before starting canal preparation, 3-dimensional, high resolution CBCT images were obtained using of Alphard-3030 dental CT system (Asahi Roentgen Co., Ltd., Kyoto, Japan). The 0.5 mm layers of images were taken axially, with 3 mm distance from the radiographic apices and perpendicular to the long axis of the roots.

The images were evaluated using the specific software (OnDemand 3D software, Cybermed Inc, Irvine, CA). A1 and B1 measurements were made using the method introduced by Gambill *et al.* [21] as follows: A1; the minimum distance between the external surface of the root section and the mesial external surface of the uncleaned root canal and B1; the minimum distance between the external surface of the root section and the distal external surface of the uncleaned root canal. The measurements were repeated three times during one week in order to increase the accuracy and calibration. The images were saved in a computer for comparison with images after canal instrumentation.

After preparation, CBCT images were taken exactly in the same manner. A2 and B2 measurements were defined as A2; the minimum distance between the external surface of the root section and the mesial external surface of the cleaned root canal and B2; the minimum distance between the external surface of the root section and the distal external surface of the cleaned root canal. The amount of transportation was calculated according to the following formula: [(A1-A2)-(B1-B2)]. After insertion of the values in the formula, if the resultant value was zero, there was no apical transportation. Any value other than zero indicated canal transportation.

Data of the two groups were evaluated by the Mann-Whitney U test and the chi-square test was used for the qualitative evaluation of transportation. The level of significance was set at 0.05.

## Results

Based on the results of the present study, the mean±SD of apical transportation with K-Flexofile and K3 instruments was 0.150±0.127 and 0.105±0.088 mm, respectively with no statistically significant differences between the two groups (*P*=0.20) (Table 1).

K-Flexofiles resulted in apical transportation in 75% of the samples; and K3 files resulted in apical transportation in 70% of the specimens. The chi-square test did not reveal any significant differences in terms of apical transportation (Table 2). 

Also the level of apical transportation in the canal was not significantly different between two groups (Table 3).

## Discussion

The present experimental study compared iatrogenic apical transportation in root canals instrumented with K3 rotary system to hand K-Flexofile. The results showed that both file systems created some apical transportation during root canal preparation; interestingly, there were no significant differences in apical transportation or its distance from the apical foramen, between the manual (K-Flexofile) and rotary systems (K3). It appears that similar to hand instrumentation, the K3 system can preserve root canal centrality.

Oliveira *et al. *[22] carried out a similar study using CBCT technology and reported no significant differences in terms of apical transportation and canal centering ability between rotary systems and hand stainless steel files. However, similar to the present study higher number of canals without transportation was obtained when using RaCe and K3 rotary instruments.

In a study by Lopez *et al. *[23] on the amount of apical transportation after canal preparation with three different file systems, hand files resulted in significantly more apical transportation while K3 system resulted in adequate safety in apical preparation and less transportation.

Guelzow *et al*. [24] compared the centering ability of six rotary systems (RaCe, ProTaper, K3, Hero 642, ProFile GT and FlexMaster) with hand files and reported that in comparison with FlexMaster and K3 files, ProTaper system resulted in greater straightening of the canals. In the present study, both manual and rotary instrumentation exhibited some amounts of apical transportation.

Using computed tomography Ozer *et al*. [25] found that using rotary systems with non-cutting tips did not reduce the apical transportation. Another similar study showed that preparation of root canals with 28‒35^°^ curvatures using K3 files, resulted in a significant decrease in root canal straightening [26]. In the study by Mokhtary *et al. *[9], BioRaCe and Mtwo rotary instruments were considered suitable for canal preparation to greater apical sizes compared to K-Flexofile provided that the recommended sequences are observed. Nazari Moghadam *et al. *[27] found that Twisted File and Reciproc systems did not differ significantly in terms of canal centering ability and transportation. Madani *et al. *[28] evaluated the root canal transportation after using two rotary systems (Mtwo & ProTaper) using CBCT and found insignificant apical transportation in both systems. Jodway and Hulsmann [29] conducted a study on canal preparation with K3 rotary files and nickel-titanium (NiTi) hand files and showed that the root canals in the K3 group exhibited some canal straightening. The differences in the results might be attributed to the various techniques used to determine the amount of canal transportation.

**Table 3:** Frequencies of apical transportation in root canals prepared with K-Flexofile and K3 instruments in different canal sections

**Group**	**Apical Transportation N (%)**					
	**Absent**	**0.1 mm**	**0.2 mm**	**0.3 mm**	**0.4 mm**	**Total**
**K-Flexofile**	5 (25)	6 (30)	5 (25)	2 (10)	2 (10)	20 (100)
**K3**	6 (30)	8 (40)	5 (25)	1 (5)	0 (0)	20 (100)
**Total**	11 (27.5)	14 (35)	10 (25)	3 (7.5)	2 (5)	40 (100)

The results of another study on extracted human teeth showed that the initial shape of canals were better preserved with the use of rotary files compared to K-Flexofile hand instruments [30]. K3 rotary files have been shown to create only a small amount of canal transportation in comparison with K-Flexofile hand instruments which showed greater transportation [26, 31]. Although the present study revealed similar results, the difference was not significant. As preparing canals with K3 rotary files is less time consuming in comparison with hand instrumentation, K3 may still be preferable.

In the present study, each file set was discarded after use in one MB canal or in case of observing any defect or deformation in the shape or structure of files. In separate studies by Akhlaghi *et al*. [32] and Lopez *et al.* [33] the files were replaced after preparation of 5 and 3 canals, respectively.

In the present study, the MB root canals of maxillary first molars were evaluated because these canals usually have significant curvatures [2]. In addition, these roots are very narrow which significantly increase the difficulty of preparing these canals [2, 21].

## Conclusion

K3 rotary system and K-Flexofile hand instruments can preserve the original canal curvature.
